# Preterm preeclampsia screening and prevention: a comprehensive approach to implementation in a real-world setting

**DOI:** 10.1186/s12884-025-07154-6

**Published:** 2025-01-15

**Authors:** Stefania Ronzoni, Shamim Rashid, Aimee Santoro, Elad Mei-Dan, Jon Barrett, Nanette Okun, Tianhua Huang

**Affiliations:** 1 Division of Maternal-Fetal Medicine, Department of Obstetrics and Gynecology, Sunnybrook Health Sciences Centre, University of Toronto, Dan Women & Babies Program, Toronto, ON Canada; 2 Genetic Program, North York General Hospital, Toronto, ON Canada; 3 Department of Obstetrics and Gynecology, North York General Hospital, University of Toronto, Toronto, ON Canada; 4 Better Outcomes Registry & Network (BORN) Ontario, Prenatal Screening Ontario, Ottawa, ON Canada

**Keywords:** Preterm preeclampsia, Prenatal screening, Low-dose acetylsalicylic acid, Placental growth factor, Uterine artery Doppler, Biomarkers, First trimester, Mean arterial blood pressure

## Abstract

**Background:**

Preeclampsia significantly impacts maternal and perinatal health. Early screening using advanced models and primary prevention with low-dose acetylsalicylic acid for high-risk populations is crucial to reduce the disease’s incidence. This study assesses the feasibility of implementing preterm preeclampsia screening and prevention by leveraging information from our current aneuploidy screening program in a real-world setting with geographic separation clinical site and laboratory analysis site.

**Methods:**

A prospective cohort study involved pregnant individuals undergoing nuchal translucency scans between 11 and 14 weeks. Risk for preterm preeclampsia was assessed using the Fetal Medicine Foundation algorithm, which includes maternal risk factors, uterine artery Doppler, mean arterial pressure and serum markers (Placental growth factor, PlGF and Pregnancy-associated plasma protein-A, PAPP-A). High-risk patients were offered low-dose acetylsalicylic acid prophylaxis. Feasibility outcomes, such as recruitment rates, protocol adherence, operational impact, integration with existing workflows, screening performance and pregnancy outcomes, were evaluated.

**Results:**

Out of 974 participants, 15.6% were deemed high-risk for preterm preeclampsia. The study achieved high recruitment (82.1%) and adherence rates, with 95.4% of high-risk patients prescribed low-dose acetylsalicylic acid. Screening performance, adjusted for low-dose acetylsalicylic acid use, showed a detection rate of 88.9–90% (FPR 13.0% and 12.7%) for preterm preeclampsia. High-risk group for preeclampsia had higher incidences of adverse outcomes, including preterm preeclampsia (7.5 vs 0.4%; *p* < 0.001), preterm delivery (21.2 vs 6.2%; *p* < 0.001), low birth weight (23.3 vs 5.6%; *p* < 0.001) and birthweight < 10th percentile (11% vs 5.6%; *p* = 0.015) compared to low-risk group. The integration of preeclampsia screening had a minimal effect on the time required for aneuploidy screening, with results obtained within a rapid turnaround time.

**Conclusions:**

The study confirms the feasibility of integrating comprehensive preeclampsia screening into clinical practice, notwithstanding geographic separation between laboratory and clinical settings. It underscores the need for broader adoption and enhanced infrastructure to optimize patient care and outcomes across diverse healthcare settings.

**Trial registration:**

Clinical trial: NCT04412681 (2020–06-02).

**Supplementary Information:**

The online version contains supplementary material available at 10.1186/s12884-025-07154-6.

## Background

Preeclampsia (PE) is a significant pregnancy-related condition affecting 2–8% of all pregnancies [1–3], leading to severe maternal and perinatal morbidity and mortality, particularly when it occurs before 32 weeks [4–6]. It also increases the risk of future cardiovascular diseases in affected individuals [7–9].

While traditional screening methods are limited in their effectiveness [10–12], recent advancements in predicting models, such as the one developed by the Fetal Medicine Foundation (FMF) in the UK, show a detection rate of approximately 75–90% of at-risk individuals in early pregnancy at a false positive rate of 10% [13, 14]. This algorithm incorporates maternal risk factors, uterine artery Doppler (UtAPI), mean arterial pressure (MAP), and serum markers of placental function (pregnancy-associated plasma protein-A (PAPP-A) and placental growth factor (PLGF) and has been validated prospectively in several studies [15–18].

Several meta-analyses have reported that the incidence of preterm PE can be substantially reduced by the administration of low-dose acetylsalicylic acid (LDA) starting at 16 weeks or earlier [19–22]. Moreover, the ASPRE trial supported the efficacy of LDA (150 mg) in high-risk pregnant individuals identified through the FMF screening algorithm, showing a significant reduction of preterm PE (83% < 34 weeks and 62% < 37 weeks) compared with placebo [23]. Several national societies support the use of LDA for the prevention of preterm PE [24, 25]. However, despite this evidence, doubts remain among practitioners about systematic screening and treatments with the result of a significant underuse of LDA in at-risk populations [26–28]. Cost-effectiveness analyses suggest that implementing screening programs and preventive measures can lead to significant cost savings for healthcare systems [29, 30].

To demonstrate that screening and treatment produce reproducible positive effects on the incidence of preterm PE in varied settings, several controlled studies of implementation have been carried out in different countries, two of them in Ontario and Alberta [31–33]. In all studies, screening was offered alongside first-trimester aneuploidy screening programs with high-risk patients advised to initiate LDA under the supervision of their healthcare provider. Both Canadian studies demonstrated the practical feasibility and efficacy of integrating an evidence-based screening and prevention program for preterm PE into clinical services where patients were screened at the clinic and laboratory tests were conducted within the same institution.

This study aims to evaluate the feasibility of implementing a clinical model for precision screening of preterm PE into the current prenatal aneuploidy screening service in a prenatal tertiary center (Sunnybrook Health Sciences Centre, Toronto, ON) in collaboration with the largest provincial prenatal screening laboratory located at a different institution (North York General Hospital, Toronto, ON).

## Methods

This prospective cohort study included singleton pregnant individuals (≥ 18 years old) undergoing nuchal translucency (NT) scan between 11 to 14 weeks at Sunnybrook Health Science Centre. Eligible individuals were offered preterm preeclampsia screening and recruited over a period of 22 non-consecutive months from March 2021 to June 2023. Exclusion criteria included fetal demise or major fetal anomalies, known contraindications or sensitivities to, or current use of LDA at the time of recruitment. The study was approved by the institutional review boards at both institutions (Sunnybrook #20–3657 and North York #20–0001) and requested to be registered Clinical.trial.gov: NCT04412681.

Upon arrival and registration at Sunnybrook Hospital ultrasound department, pregnant individuals registered for an NT scan received an information pamphlet about the study (*Suppl Fig. 1*) from the ultrasound check-in clerk. As per implemented department protocol, the NT scan included uterine artery Doppler by certified sonographers who had previously obtained the FMF Certificate of Competence [34]. Uterine artery Doppler pulsatility index (UtAPI) was measured from the left and right sides using criteria in accordance with the standardized FMF protocol [35]. After the ultrasound, patients were approached by a designated research associate who invited them to participate in the implementation study. Interested patients who met eligibility criteria received comprehensive information about the study and were given the opportunity to sign informed consent. In consenting study participants, the research associate recorded maternal demographic and risk factors required for preterm PE risk calculation as reported by participants. Maternal blood pressure was measured in a quiet room with a validated automated device according to the standardized FMF protocol [36]. These data were recorded on a standard screening requisition for PE (*Suppl Fig. 2*). All participants planning to undergo aneuploidy screening were then directed to the hospital laboratory or external laboratory to obtain a blood sample within a couple of days after ultrasound, following standard practice for first-trimester screening (FTS). In Ontario, standard screening for aneuploidy includes four serum markers (AFP, PAPP-A, PlGF, and free BHCG). The preterm PE screening requisition was added to the current aneuploidy screening form. All samples were sent to the provincial prenatal screening laboratory (North York General Hospital).

Additionally, participants who were interested in PE screening only (either declining aneuploidy screening or having already had noninvasive prenatal testing, NIPT) were given the option to undergo the blood test in the laboratory. In Ontario, the 11 to 14-week scan with NT measurement is recommended for all, including those declining risk calculation for aneuploidy and those having had NIPT, due to its demonstrated independent benefits [37]. In these cases, the research assistant indicated “only PE screening, no FTS” on the requisition form. Alternatively, participants had the choice to omit placental serum marker level from the risk calculation, with the understanding that this would reduce the accuracy of the test [13]. To track and verify an efficient flow of requisition forms and samples to the centralized laboratory located at North York General Hospital, the designated research assistant emailed the list of participants daily to the laboratory. The personalized risk for preterm PE was calculated at the North York General Hospital laboratory using LifeCycle (Revvity Inc.) screening software with the incorporated FMF algorithm [34]. A risk cut-off of ≥ 1/100 was employed to define high-risk patients. The majority of pregnant individuals in the study had undergone multiple marker screening for aneuploidies. The MoM values from this screening were extracted and entered into LifeCycle for the calculation of preeclampsia risk. These MoM values were generated using the Alpha screening software, which is used for routine aneuploidy screening. The MoM values were adjusted for race, smoking, insulin-dependent diabetes mellitus, and IVF. No additional adjustments were made in the PE risk calculation within the LifeCycle software. Ontario, along with screening laboratories, follows routine quality assurance (QA) processes to evaluate the measurements of each biochemical marker, including both internal lab reviews and monthly provincial QA assessments. The results of the PE screening were transmitted by fax to the research assistant at Sunnybrook Hospital. A low-risk result was communicated to the participant by phone by the research assistant, and a copy of the report was sent to the primary healthcare provider. In the event of high-risk results, either a maternal–fetal medicine specialist or an implementation study nurse practitioner was informed by the research assistant. They were responsible for providing adequate counselling by phone to the patient, including advice to commence LDA (162 mg) prior to 16 weeks until 36 weeks (as per local standard of care). A copy of the high-risk results and suggested pregnancy management (LDA, placental study, serial fetal growth assessment, fetal medicine specialist consultation) was sent to the primary health care provider, responsible for further management. LDA adherence was monitored through patient’s self-report during serial follow-up phone calls conducted by the research assistant at 16, 22, 26, 32, and 36 weeks. Adherence data were captured as a binary response (yes/no) to taking LDA for each call, and reasons for discontinuation were documented.

Participants’ demographic information, screening data, risk estimation for preterm PE, interventions, and LDA adherence were entered into the study database in real time by the research coordinators. Pregnancy outcomes, including preterm PE, late PE, preterm birth at less than 34 and 37 weeks, stillbirth, low birth weight (< 2500 g) and small for gestational age (SGA) birthweight (< 10th and < 3rd percentile derived via Intergrowth-21st from gestational age and sex at birth [38]) were collected by chart review after delivery and entered by the research coordinator and verified in the study database by clinical researchers.

Quality assurance of placental growth factor (PLGF) and pregnancy-associated plasma protein A (PAPP-A) measurements are routinely evaluated as part of the quality assurance for multiple marker screening. Quality assurance of uterine artery pulsatility index and blood pressure measurements were monitored by assessing monthly and operator-specific medians against the FMF nomograms. PE and gestational hypertension were defined according to the International Society for the Study of Hypertension in Pregnancy [39]. Preterm PE was defined as delivery with PE before 37 weeks of gestation and did not include gestational hypertension. The screen detection rate (sensitivity) and false positive rate (1-specificity) for preterm PE < 37 weeks was calculated, including estimates adjusted for the effect of LDA in high-risk patients. The detection rates and false positive rates, after adjusting for LDA use, were calculated under the assumption that LDA use prevented 62% of preterm PE cases [23]. Specifically, the expected number of PE cases was calculated as the sum of number of PE cases with low risk and number of PE cases with high risk divided by adherence rate multiplied by 62%. Expected number of cases predicted was calculated by the number of PE cases with high risk divided by adherence rate multiplied by 62% [33, 40]. We used both the highest and lowest LDA adherence rates observed in the study, along with a range of hypothetical LDA adherence rates that might be seen in a screening population. The Mann–Whitney U test, Chi-Square test, and Fisher's Exact test were utilized to compare the maternal characteristics and prevalence of adverse outcomes between the high-risk and low-risk groups. The sample size was estimated based on the assumption that screening 1,000 patients for preterm PE would be adequate to evaluate the operational and logistical aspects of implementation. With a 1 in 100 cutoff, this would result in approximately 100 screen-positive pregnancies (at least 10%).

## Results

**Fig. 1:**
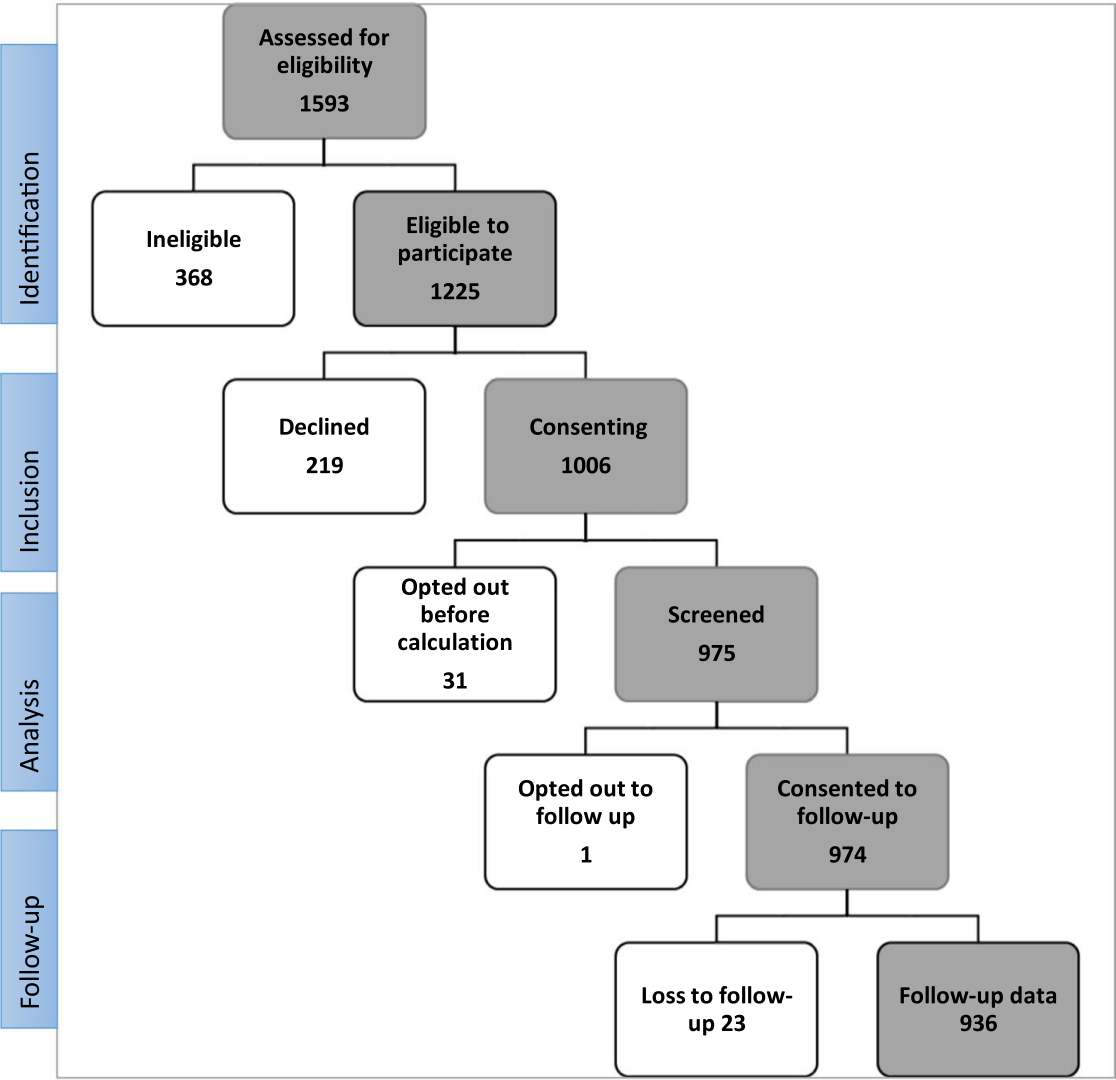
Pre-eclampsia screening study inclusion flow diagram

Out of 1225 eligible patients, 1006 consented to participate in the study (82.1%). The recruitment rate demonstrated an improvement throughout the study, rising from 74.3% to 94.3% upon the inclusion of patients opting for NIPT, allowing for the choice to calculate PE risk with or without the serum markers. Among 1006 consenting patients, 31 opted out before and 1 patient after the PE risk calculation (32/1006, 3.2%), resulting in a final cohort of 974 patients (Fig. 1).

PE screening identified 152 patients as being at high risk (15.6%) and 822 at low risk (84.4%). PE risk was calculated without the use of placental markers in 157 out of 974 patients (16.1%), which have been included in the analysis.

Table 1 provides the demographic characteristics, medical and obstetrical history of enrolled patients subdivided into low and high-risk groups based on PE screening results. Patients identified as high-risk were more likely to be nulliparous, Black or South-Asian, smokers, conceiving through in vitro-fertilization, or had longer interpregnancy intervals if multiparous, had a higher BMI and medical complications such as chronic hypertension, autoimmune conditions and diabetes.

**Table 1:** Demographics, medical and obstetrical history of the study population

Demographics, Obstetrics and History	Low Risk Median[IQR]/N(%)	High risk Group Median[IQR]/N(%)	Total Median[IQR]/N(%)	*P*-value
Number of patients	*N* = 822	*N* = 152	*N* = 974	-
Maternal age at due date (years)	34.3 [31.5–36.7]	34.6 [31.4–37.7]	34.3 [31.4–36.9]	0.60
Gestational age at screen (weeks)	12.3 [12.0–12.5]	12.2 [12.0–12.5]	12.3 [12.0–12.5]	0.20
Body mass index	24.1 [21.6–27.5]	28.1 [23.8–34.0]	24.5 [21.8–28.4]	** < 0.0001**
Race or ethnic group^a^				
White	438 (53.3)	49 (32.2)	487 (50.0)	Reference
Black	35 (4.3)	25 (16.4)	60 (6.2)	** < 0.0001**
East Asian	167 (20.3)	28 (18.4)	195 (20.0)	0.11
South Asian	129 (15.7)	39 (25.7)	168 (17.2)	** < 0.0001**
Other	53 (6.4)	11 (7.2)	64 (6.6)	0.09
Total	822 (84.4)	152 (15.6)	974 (100)	-
Method of conception				
Spontaneous	785 (95.5)	132 (86.8)	917 (94.1)	Reference
In vitro fertilization	37 (4.5)	20 (13.2)	57 (5.9)	** < 0.0001**
Total	822 (84.4)	152 (15.6)	974 (100)	-
Medical History				
Chronic hypertension	6 (0.7)	25 (16.4)	31 (3.2)	** < 0.0001**
Systemic lupus erythematosus	3 (0.4)	3 (2.0)	6 (0.6)	0.052
Antiphospholipid syndrome	-	1 (0.7)	1 (0.1)	Insufficient count
Diabetes mellitus type 1	6 (0.7)	4 (2.6)	10 (1.0)	0.056
Diabetes mellitus type 2	10 (1.2)	15 (9.9)	25 (2.6)	** < 0.0001**
Cigarette smoking during pregnancy	9 (1.1)	6 (3.9)	15 (1.5)	** < 0.0194**
Parity				
Nulliparous	379 (46.1)	96 (63.2)	475 (48.8)	Reference
Multiparous	443 (53.9)	56 (36.8)	499 (51.2)	** < 0.0001**
Total	822 (84.4)	152 (15.6)	974(100)	**-**
Obstetric and Family History				
History of preeclampsia	4 (0.9)	12 (21.4)	16 (1.6)	** < 0.0001**
Mother had preeclampsia	18 (2.2)	6 (3.9)	24 (2.5)	0.2452
Interval from last pregnancy (years)	2.1 [1.4–3.4]	2.95 [1.9–4.9]	2.2 [1.4–3.5]	** < 0.01**

^a^All races are compared to the white reference group

The turnaround time between data screening collection, blood sample collection in any laboratory and result reporting was about 2 days. The individual time intervals for each screening component, including history collection, blood pressure measurement, uterine artery Doppler measurement and overall screening process till results are detailed in Table 2.

**Table 2:** Time used for each component of the screening test and turnaround time of the screening test

Time Interval	Median (p5, p95)
History Collection (min)	2.0 (1.0, 4.25)
Blood Pressure Collection (min)	3.0 (2.0, 4.0)
Nuchal Translucency Scan (min)	25 (20,40)
Uterine artery Doppler measurement (min)	2.0 (1.0,2.5)
Time between blood sample collection and blood sample receipt (days)	1(1,2)
Time between sample receipt and results report (days)	2(1,3)

Pregnancy outcomes were available in 936 cases (96.1%), (790 low risk and 146 high risk) with 23 loss of follow-up (delivered in another hospital), 8 terminations of pregnancy before 24 weeks, 5 miscarriages < 16 weeks, and 2 intrauterine deaths < 20 weeks and are presented in Table 3.

**Table 3:** Pregnancy outcomes among pregnancies > 20 weeks’ gestation with high or low risks for preeclampsia

Pregnancy outcomes	Low risk N (%)	High risk N (%)	Total N (%)	*P* value
Number of patients	790 (84.4)	146 (15.6)	936 (100)	-
Preeclampsia < 37 weeks	3 (0.4)	11 (7.5)	14 (1.5)	** < 0.001**
Preeclampsia < 34 weeks	1 (0.1)	-	1 (0.1)	-
Preterm delivery < 37 weeks	49 (6.2)	31 (21.2)	80 (8.5)	** < 0.001**
Preterm delivery < 34 weeks	17 (2.2)	10 (6.8)	27 (3.0)	** < 0.006**
Birthweight < 2500 g	44 (5.6)	34 (23.2)	78 (8.4)	** < 0.001**
Stillbirth	-	1 (0.7)	1 (0.1)	-
Birthweight < 10%tile	44 (5.6)	16 (11)	60 (6.4)	= 0.015
Birthweight < 3%tile	9 (1.1)	2 (1.4)	11(1.2)	= 0.812

The high-risk subgroup presented with significantly higher rates of adverse pregnancy outcomes, including preterm PE < 37 weeks (7.5% vs 0.4% *p* < 0.001), preterm delivery (21.2% vs 6.2% *p* < 0.001), birthweight < 2500 g (23.2% vs 5.6% *p* < 0.001), and < 10%tile (11% vs 5.6% *p* = 0.015) (Table 3). Additionally, among high-risk patients, there were 3 cases of PE after 37 weeks (2.0%) and one postpartum PE requiring readmission (0.7%). In the low-risk group, there were 5 cases of post-partum PE requiring readmission (0.6%).

When compared with low-risk, high-risk patients had significantly higher median MAP (1.03 [0.92–1.2] vs 0.97 [0.87–1.11] MoM, *p* < 0.001), UtAPI (1.19 [0.75–1.78] vs 0.99 [0.60–1.45] MoM; *p* < 0.001) and lower PLGF (0.76 [0.24–1.35] vs 1.08 [0.56–1.94] MoM; *p* < 0.001), and lower PAPP-A ( 0.89 [0.30–2.09] vs 1.12 [0.48–2.64] MoM; *p* < 0.001) (Table 4).

**Table 4:** Median MoM of biophysical and biochemical marker by pregnancy outcomes and screening results

Outcome/result	**Marker Medians (p5, p95)**				
	**N (%)**	**MAP MoM**	**UTPI MoM**	**PIGF MoM**	**PAPPA MoM**
**Adverse outcomes**					
Preterm PE < 37 weeks	14 (1.5)	1.08 (0.94,1.36)	1.00 (0.54,1.84)	0.89 (0.70,2.02)	1.14 (0.30,2.24)
Preterm delivery < 37 weeks	80 (8.5)	1.01 (0.86,1.21)	1.08 (0.62,1.62)	1.04 (0.46,1.97)	1.08 (0.33,2.53)
Preterm delivery < 34 weeks	27 (2.9)	1.00 (0.87,1.15)	1.11 (0.62,1.54)	1.02 (0.45,1.65)	0.97 (0.37,1.96)
Gestational hypertension	22 (2.4)	1.06 (0.94,1.19)	1.15 (0.64,1.44)	0.86 (0.53,2.38)	0.86 (0.36,3.11)
Birthweight < 2500 g	78 (8.3)	1.01 (0.90,1.17)	1.14 (0.58,1.71)	0.89 (0.45,1.85)	0.91 (0.33,2.43)
All adverse outcomes	208 (22.2)	1.01 (0.87,1.17)	1.05 (0.62,1.62)	0.97 (0.46,2.02)	0.99 (0.36,2.53)
**Screening result**					
All pregnancies	974 (100)	0.98 (0.87,1.13)	1.01 (0.62,1.52)	1.04 (0.46,1.90)	1.09 (0.41,2.58)
Positive PE Screen	152 (15.6)	1.03 (0.92,1.20)	1.19 (0.75,1.78)	0.76 (0.24,1.35)	0.89 (0.30,2.09)
Negative PE Screen	822 (84.4)	0.97 (0.87,1.11)	0.99 (0.60,1.45)	1.08 (0.56,1.94)	1.12 (0.48,2.64)

*PE* Pre-eclampsia, *MAP* Mean arterial pressure, *UTPI* Uterine arteries pulsatility index, *PlGF* Placental Growth Factor, *PAPP-A* Pregnancy-Associated Plasma Protein A, *MoM* Multiple of the median

Telephone follow-up of high-risk patients was successful in all high-risk pregnancies until 26 weeks, with three patients lost to follow-up (2.1%) thereafter. The initiation rate of LDA at 16 weeks was 95.4% (144/151), with only 7 patients not commencing LDA due to personal choice or following medical advice in the presence of antepartum bleeding (Table 5).

**Table 5:** Compliance to low dose acetylsalicylic acid (LDA) in high-risk patients: initiation and maintenance rate

Check in Week	Taking LDA	Not Taking LDA	Miscarriage	Loss Follow Up	Delivery	Total Pregnancies **
	**N (%)**	**N (%)**	**N (%)**	**N (%)**	**N (%)**	**N**
16	144 (95.4)	7 (4.6)	1 (0.7)	0 (0)	0 (0)	151
22	138 (92.6)	11 (7.4)	2 (1.3)	0 (0)	1 (0.7)	149
26	135 (91.2)	13 (8.8)	2 (1.4)	0 (0)	2 (1.4)	148
32	130 (90.9)	13 (8.9)	2 (1.4)	3 (2.1)	4 (2.8)	143
36	111 (86.0)	18 (14.0) ^a^	2 (1.6)	3 (2.3)	18 (14.0)	129

^a^5 patients stopped taking LDA acid at 35 weeks

**The number of pregnancies reported at each gestational time point reflects those that have reached that stage of pregnancy (excluding those who had terminated, delivered or lost follow-up). LDA low-dose acetylsalicylic acid

Among 11 high-risk patients who developed preterm PE, all were compliant with LDA until delivery. In 6 cases, PE was superimposed on chronic hypertension, with 3 cases associated with preexisting severe chronic kidney disease and 1 case with autoimmune kidney disease. One patient with a prior history of severe preterm PE at 26 weeks developed PE at 36.5 weeks. In the low-risk population, one case of preterm PE (< 34 weeks) occurred in a patient with a severe renal pathology who was on LDA despite low-risk PE screening results. None of the other 4 low-risk cases diagnosed with PE were on LDA. In this study, 11 of the 16 (68.8%) observed preterm PE screened had high-risk screening results.

Treatment-adjusted screening performance for preterm PE at different LDA adherence rates is shown in Table 6. In this study, the LDA adherence rates range between 86% and 95.4% at different gestational ages. With these adherence rates, the DR adjusted for LDA use were 88.9% and 90.0% for FPR of 13.0% and 12.7% respectively.

**Table 6:** Screen performance at different LDA adherence thresholds

**Disease frequency and screening performance**	**Study LDA adherence**^**a**^		**Hypothetical LDA adherence**^**b**^					
	86.0%	95.4%	50%	60%	70%	80%	90%	100%
Expected PE reduction %^c^	53.3%	59.1%	31.0%	37.2%	43.4%	49.6%	55.8%	62.0%
Expected total number of cases^d^	27	30	19	21	22	25	28	32
Expected detected cases^e^	24	27	16	18	19	22	25	29
Adjusted detection rate	88.9%	90.0%	84.2%	85.7%	86.4%	88.0%	89.3%	90.6%
Adjusted false positive rate	13.0% (146-24)/936	12.7% (146-27)/936	13.9%	13.7%	13.6%	13.2%	12.9%	12.5%

Calculated based on 146 high-risk pregnancies and 14 preterm preeclampsia cases observed in the study

^a^Based on the lowest and highest self-reported adherence rates in high-risk patients participating in follow-up at 16, 22, 26, 32 and 36 weeks' gestation

^b^Hypothetical rates that we consider that might be seen in a screening population

^c^Assuming LDA use has prevented 62% of the preterm preeclampsia cases

^d^Sum of cases from low-risk group, high-risk group and potential cases from the high-risk group that have been prevented by LDA

^e^Sum of cases from the high-risk group and potential cases from the high-risk group that have been prevented by LDA (calculated based on 3 low cases and 11 high-risk cases)

## Discussion

This study demonstrates the feasibility of implementing first-trimester aneuploidy screening and preterm PE screening in a real-world clinical setting, with PE risk calculation conducted at a separate site from the clinical setting. High enrollment rates and strong patient participation underscore the practicality and effectiveness of integrating comprehensive screening protocols into diverse healthcare settings with an ultrasound facility-based program. Although not powered to fully evaluate maternal and fetal outcomes, the study indicates improved preterm PE detection rates and enhanced adherence to LDA among high-risk patients compared to conventional screening.

The results of this study align with two previous studies on the implementation of preterm PE screening programs in Canada (*Suppl Table 1*). The studies reported similar metrics in the enrollment rate, particularly when we also considered eligible patients who had aneuploidy risk evaluated by NIPT before performing the NT scan. Similar to the routine multiple marker aneuploidy screening, our study had a spatial separation between the laboratory and clinical settings. However, we found minimal operational impact comparable to the two Canadian studies where recruitment, clinical assessment, and laboratory work were conducted at a single site [32, 33]. Although our study did not specifically evaluate patient satisfaction as the other two studies have reported, the completion rate for follow-up was notably high and comparable to the other Canadian studies reviewed.

Our screen-positive rate was higher (15%) than the 8.2% and 10.4% reported in the two Canadian studies [32, 33] but consistent with findings from larger studies evaluating the performance of the FMF algorithm [41]. This discrepancy could be attributed to several factors, including differences in the screened population, where we included higher-risk patients and variations in patients' race or ethnic group. Our study also demonstrated the highest estimated detection rate of 90% with theoretical 100% LDA adherence compared to the rate reported in the other two studies (76.8% and 86.6%) [32, 33]. In this study, we did not account for the effect of LDA on the low-risk group, even though some individuals were prescribed the medication for other indications.

A recent Ontario Health (Quality) health technology assessment concluded that the FMF risk calculation model was more accurate than current clinical factor-based screening, was potentially feasible to incorporate into the current aneuploidy screening program and was potentially cost-effective. The assessment reported that patients value both access to a screening and preventive approach and the increased information available regarding PE and placental dysfunction [42]. A significant aspect of our study lies in the practical integration of preterm PE screening into routine clinical practice, closely mirroring real-world conditions in the laboratory and clinical settings. While the FMF algorithm provides better performance when all parameters (UtAPI, PlGF, and PAPP-A) are included, it can still deliver a personalized risk score even if one or both placental markers are missing. This makes the protocol applicable to lower-resource settings where not all parameters may be available.

This enhances the potential generalizability and scalability of our findings, offering a more accurate representation of potential implementation outcomes in broader clinical settings. This study has some limitations. First, some patients delivered at other institutions, leading to a loss of follow-up data. Additionally, the collection of race-based data was limited to specific categories in the risk calculation software. We are aware of the lack of data on level of risk or differences in biochemical or biophysical markers in a broader range of race categories. More importantly, we are obligated to explore whether these differences are related to intersecting confounding features such as racism or socio-demographic differences [32]. Furthermore, while we integrated preterm PE screening into the routine enhanced first-trimester screening process, we acknowledge that race-based categories in risk assessment may not fully reflect the biological and genetic diversity of the population, which should be considered when interpreting our findings. In addition, in this study, the MoM value of serum markers was not calculated using the PE screening software, but we utilized the measurement already available from the aneuploidy screening. While we acknowledge that it is optimal to use a single software for both screening tests, we were able to demonstrate how to leverage information already available from aneuploidy screening for preeclampsia screening in the study. Moreover, compliance with LDA was based on patient self-report, as LDA is available over the counter and could not be audited by pharmacy records. While we could not completely verify medication access or adherence, the affordability of LDA suggests that compliance was likely reliable. Finally, a limitation that should be considered is potential barriers to the generalizability of the study due to the lack of facilities equipped to measure placental growth factors or uterine arteries dopplers, which restrict the widespread application of the protocol. Additionally, the strict methodology used for blood pressure measurement, including controlled conditions like a quiet room and measurements from both arms, may not reflect routine clinical practice, where such conditions are not always feasible. This should be considered when interpreting the applicability of our findings to everyday clinical settings.

Validated modifications to screening algorithms, albeit excluding certain components, offer viable options to enhance accessibility while maintaining a detection rate superior to clinical risk factor-based screening [43]. Moving forward, addressing these barriers through enhanced training, expanded infrastructure, and improved healthcare provider engagement could further optimize the adoption and impact of our screening approach across diverse healthcare settings.

## Conclusions

This study successfully demonstrated the feasibility of implementing a preterm PE multiple marker screening and prevention program in a clinical setting. The findings highlight the potential for broader adoption of such programs, which could lead to significant reductions in the adverse impacts of preterm PE among pregnant individuals and their babies. Support for systematic PE screening programs is growing, emphasizing the importance of high-quality implementation and the potential benefits for patients, healthcare providers, and the healthcare system overall. Future research should prioritize scaling up preterm PE screening programs by directly involving primary care providers and centralizing data collection and calculations and quality assurance assessments akin to successful models used in aneuploidy screening. Such efforts are crucial for optimizing patient care, improving outcomes, and fostering broader adoption of effective screening strategies in prenatal healthcare.

## Supplementary Information

Supplementary Material 1. Supplementary Table 1: Comparison of the current study with two similar published Canadian implementation studies.

Supplementary Material 2. Supplementary Figure 1: Information pamphlet about the study.

Supplementary Material 3. Supplementary Figure 2: Standard Preeclampsia screening requisition.

## Data Availability

No datasets were generated or analysed during the current study.

## Abbreviations

PE: Preeclampsia
LDA: Low dose acetylsalicylic acid
NT: Nuchal translucency
UtAPI: Uterine arteries pulsatility Index
MAP: Mean arterial blood pressure
PAPP-A: Pregnancy-associated plasma protein-A
PLGF: Placental growth factor
FMF: Fetal Medicine Fundation
FTS: First trimester screening
NIPT: Noninvasive prenatal testing
