# Supplementary material for: Preterm preeclampsia screening and prevention: a comprehensive approach to implementation in a real-world setting
Source: BMC Pregnancy Childbirth. 2025 Jan 15;25:32. doi: 10.1186/s12884-025-07154-6 (PMC11734365; doi:10.1186/s12884-025-07154-6)
Supplement: Supplementary file 1 — Supplementary Material 1. Supplementary Table 1: Comparison of the current study with two similar published Canadian implementation studies. [file 12884_2025_7154_MOESM1_ESM.docx]

**Supplementary Table 1: Comparison of the current study with two similar published Canadian implementation studies**

|  | Current study | Johnson et al  AJOG MFM, 2023 | Okun et al,  JOGC 2023 |
| --- | --- | --- | --- |
| Eligible to participate (#) | 1225 | 1142 | 1247 |
| Enrolled (consent signed) | 1006/1225  (82.1%) | 1124/1142  (98.4%) | 1090/1247  (84.7%) |
| Screened | 975/1006  (96.8%) | 1124/1124  (100%) | 1057/1090  (96.9%) |
| Completed follow up | 974/975  (99.9%) | 1119/1124  (99.5%) | 931/1057  (88.1%) |
| History collection/Mean Arterial Pressure (min) | 5.47 | 6.0 | 10.5 |
| Uterine arteries measurements (min) | 2.02 | 2.0 | 3.4 |
| Sample collection, sample receiving (days) | 1.42 | - | - |
| Sample receiving to result reporting (days) | 1.65 | - | - |
| Sample collection to result reporting (days) | 3.07 | - | - |
| Screen positive rate for preeclampsia<37 weeks (n/%) | 152  15.3% | 96  8.2% | 110  10.4% |
| Initiation LDA at 16 weeks (%) | 95.4% | 87% | 88.9% |

LDA: low dose acetylsalicylic acid
