# Supplementary material for: Preterm preeclampsia screening and prevention: a comprehensive approach to implementation in a real-world setting
Source: BMC Pregnancy Childbirth. 2025 Jan 15;25:32. doi: 10.1186/s12884-025-07154-6 (PMC11734365; doi:10.1186/s12884-025-07154-6)
Supplement: Supplementary file 2 — Supplementary Material 2. Supplementary Figure 1: Information pamphlet about the study. [file 12884_2025_7154_MOESM2_ESM.pdf]

What is Pre-eclampsia?

Pre-eclampsia is a specific complication of pregnancy characterized by high blood pressure. It may also involve other organs, such as the liver and/or kidneys. Pre-eclampsia can lead to serious complicatons for both mom and baby.

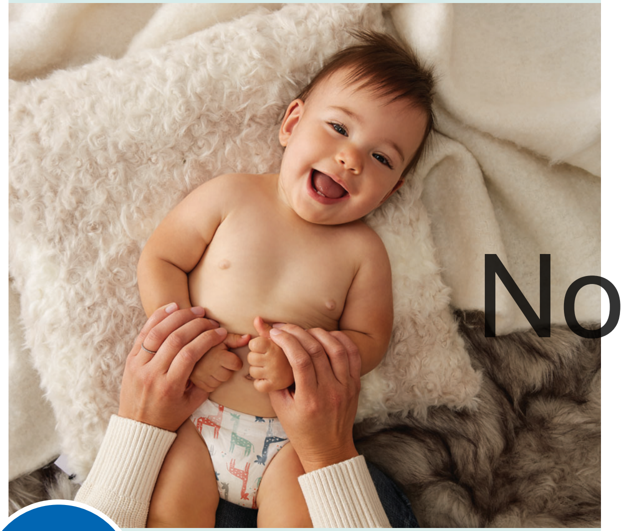

What is the cause of Pre-eclampsia?

The exact cause of pre-eclampsia remains unknown, but there is good evidence that the placenta contributes to the development of pre-eclampsia. If you want to know more information about pre-eclampsia please visit this website: preeclampsia.org/health-information

If you are interested in participating in this study, you will find our research team at the front desk

Not for reproduction

Contact information

Project Lead

Dr. Stefania Ronzoni

Sunnybrook Health Sciences Centre
2075 Bayview Avenue
Toronto, Ontario M5N 3M5
Prevention.SHSC@sunnybrook.ca

Sunnybrook
DAN WOMEN & BABIES PROGRAM

PR 60644 (December 2020)

Using Precision Medicine for the Prediction and Prevention of Early Pre-Eclampsia in Ontario

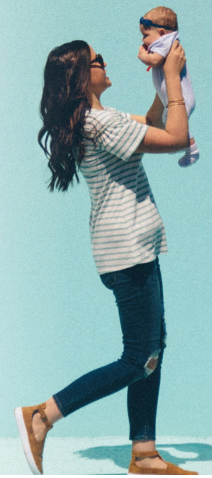

Sunnybrook
DAN WOMEN & BABIES PROGRAM

Why is this study necessary?

Before expanding a screening program to all Ontarians, we need to start in experienced centres such as Sunnybrook Health Sciences Centre to understand how best to incorporate this test into current practice.

What is the treatment for Pre-eclampsia?

The best treatment is delivery but when pre-eclampsia occurs too early in pregnancy, high blood pressure can be treated with medications. Unfortunately, medications do not always work and preterm delivery is the only option to decrease severe complications affecting maternal health. However this may have a severe impact on the health of your baby.

Is there a way to prevent Pre-eclampsia?

International research has shown that low dose aspirin, a cheap and safe medication, started in the second trimester can considerably reduce the occurrence of early pre-eclampsia in women at high risk for pre-eclampsia.

How do I Know if I am at Risk of Pre-Eclampsia?

It is now possible to estimate the risk of developing early preeclampsia as early as the 11th week of pregnancy. This test can be done at the same time as your routine 11-14 weeks ultrasound.

The test involves the following:

- Measurement of your blood pressure
- Ultrasound measurement of the blood flow of your uterine arteries
- Collection of some medical information
- Sunnybrook Health Sciences Centre is one of the first hospitals in Canada to initiate this new screening and prevention program.

Not for reproduction

Sunnybrook Health Sciences Centre is one of the first hospitals in Canada to initiate this new screening and prevention program.

The DAN Women & Babies program at Sunnybrook is supporting this research study to create a local model for implementing province-wide screening.

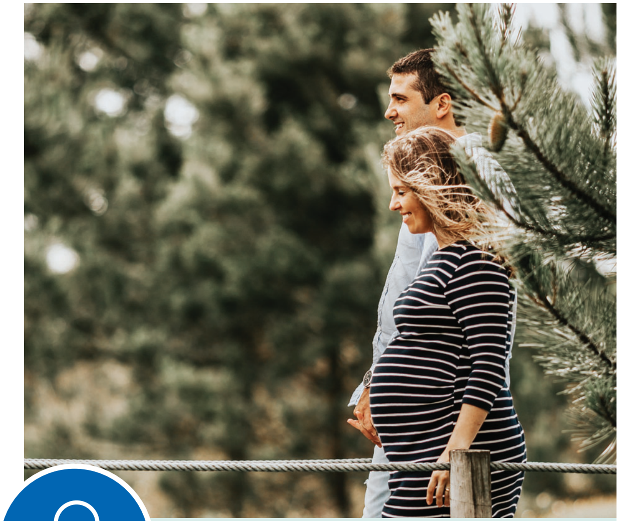

Who Can Participate in the Study?

- Pregnant women over the age of 18
- Between 11 and 14 weeks of pregnancy
- Having an ultrasound at 11 to 14 weeks of pregnancy
- Planning to deliver at Sunnybrook Health Sciences Centre

Please consider participating in the study! Being screened for pre-eclampsia is an important step you can take to protect the health of both yourself and your baby

Participation of this study is entirely your choice and you are free to withdraw for any reason at any tme.
