# Supplementary material for: Preterm preeclampsia screening and prevention: a comprehensive approach to implementation in a real-world setting
Source: BMC Pregnancy Childbirth. 2025 Jan 15;25:32. doi: 10.1186/s12884-025-07154-6 (PMC11734365; doi:10.1186/s12884-025-07154-6)
Supplement: Supplementary file 3 — Supplementary Material 3. Supplementary Figure 2: Standard Preeclampsia screening requisition. [file 12884_2025_7154_MOESM3_ESM.pdf]

Supplementary Figure 2: Pre-eclampsia screening form (additional information added to eFTS provincial form

| For PE screening calculation | Results | |
|---|---|---|
| Maternal data (demographic, history) | | |
| Maternal Height | _________cm | |
| Patient's Mother with preeclampsia | ☐ yes ☐ no | |
| Patient's history of CHTN | ☐ yes ☐ no | |
| Patient's history of LES | ☐ yes ☐ no | |
| Patient's history of APLS | ☐ yes ☐ no | |
| Patient's history of Type 1 diabetes<br>Patient's history of Type 2 diabetes | ☐ yes ☐ no<br>☐ yes ☐ no | |
| Nulliparous (no previous pregnancy ≥ 24 weeks) | ☐ yes ☐ no | |
| Parous (at least one pregnancy ≥ 24 weeks)<br>If yes:<br>• Preeclampsia in previous pregnancy<br>• Date of delivery<br>• GA at delivery<br>• Inter-pregnancy interval<br>• Birthweight | ☐ yes ☐ no<br><br>☐ yes ☐ no<br>_____/____/_____ (y/m/d)<br>_____________weeks<br>_____________years<br>___________g | |
| Maternal Blood Pressure | | |
| Measurement 1 | Left arm | Right arm |
| Systolic | | |
| Diastolic | | |
| Measurement 2 | Left arm | Right arm |
| Systolic | | |
| Diastolic | | |
| Maternal Uterine Doppler | | |
| Ut PI | Left uterine | Right uterine |
| | | |
